# High Prevalence of Insulin Resistance in Asymptomatic Patients with Acute Intermittent Porphyria and Liver-Targeted Insulin as a Novel Therapeutic Approach

**DOI:** 10.3390/biomedicines9030255

**Published:** 2021-03-05

**Authors:** Isabel Solares, Laura Izquierdo-Sánchez, Montserrat Morales-Conejo, Daniel Jericó, Francisco Javier Castelbón, Karol Marcela Córdoba, Ana Sampedro, Carlos Lumbreras, María Jesús Moreno-Aliaga, Rafael Enríquez de Salamanca, Pedro Berraondo, Antonio Fontanellas

**Affiliations:** 1 Reference Center for Inherited Metabolic Disease-MetabERN, Department of Internal Medicine, University Hospital 12 de Octubre, UCM, 28041 Madrid, Spain; isolares@alumni.unav.es (I.S.); montserrat.morales@salud.madrid.org (M.M.-C.); fjcastelbon@yahoo.es (F.J.C.); clumbrerasb@gmail.com (C.L.); salamanca@med.ucm.es (R.E.d.S.); 2 Hepatology Program, Cima Universidad de Navarra, 31008 Pamplona, Spain; laura.izquierdo@biodonostia.org (L.I.-S.); djerico@alumni.unav.es (D.J.); kcordoba@alumni.unav.es (K.M.C.); asampedro@unav.es (A.S.); 3 Department of Liver and Gastrointestinal Diseases, Biodonostia Health Research Institute, Donostia University Hospital, University of the Basque Country (UPV/EHU), 20014 San Sebastian, Spain; 4 Grupo de Enfermedades Mitocondriales y Neuromusculares, Instituto de Investigación Hospital 12 de Octubre (i+12), Centro de Investigación Biomédica en Red de Enfermedades Raras (CIBERER), Instituto de Salud Carlos III, 28029 Madrid, Spain; 5 Instituto de Investigación Sanitaria de Navarra (IdiSNA), 31008 Pamplona, Spain; mjmoreno@unav.es (M.J.M.-A.); pberraondol@unav.es (P.B.); 6 Center for Nutrition Research and Department of Nutrition, Food Science and Physiology, University of Navarra, 31008 Pamplona, Spain; 7 CIBERobn Physiopathology of Obesity and Nutrition, Carlos III Health Institute, 28029 Madrid, Spain; 8 Program of Immunology and Immunotherapy, Cima Universidad de Navarra, 31008 Pamplona, Spain; 9 Centro de Investigación Biomédica en Red de Cáncer, CIBERonc, Instituto de Salud Carlos III, 28029 Madrid, Spain; 10 Centro de Investigación Biomédica en Red de Enfermedades Hepáticas y Digestivas (CIBERehd), Instituto de Salud Carlos III, 28029 Madrid, Spain

**Keywords:** acute intermittent porphyria, carbohydrate loading therapy, insulin resistance, hyperinsulinemia, fast-acting insulin, experimental liver-targeted insulin

## Abstract

Acute porphyria attacks are associated with the strong up-regulation of hepatic heme synthesis and over-production of neurotoxic heme precursors. First-line therapy is based on carbohydrate loading. However, altered glucose homeostasis could affect its efficacy. Our first aim was to investigate the prevalence of insulin resistance (IR) in an observational case-control study including 44 Spanish patients with acute intermittent porphyria (AIP) and 55 age-, gender- and BMI-matched control volunteers. Eight patients (18.2%) and one control (2.3%, *p* = 0.01) showed a high HOMA-IR index (cut-off ≥ 3.4). Patients with IR and hyperinsulinemia showed clinically stable disease. Thus, the second aim was to evaluate the effect of the co-administration of glucose and a fast-acting or new liver-targeted insulin (the fusion protein of insulin and apolipoprotein A-I, Ins-ApoAI) in AIP mice. The combination of glucose and the Ins-ApoAI promoted partial but sustained protection against hepatic heme synthesis up-regulation compared with glucose alone or co-injected with fast-acting insulin. In a prevention study, Ins-ApoAI improved symptoms associated with a phenobarbital-induced attack but maintained high porphyrin precursor excretion, probably due to the induction of hepatic mitochondrial biogenesis mediated by apolipoprotein A-I. In conclusion, a high prevalence of IR and hyperinsulinemia was observed in patients with AIP. The experimental data provide proof-of-concept for liver-targeted insulin as a way of enhancing glucose therapy for AIP.

## 1. Introduction

Acute intermittent porphyria (AIP, MIM 176000) is an autosomal dominant metabolic disease caused by a partial deficiency of the hepatic porphobilinogen deaminase (PBGD, EC 4.3.1.8), the third enzyme of the heme synthesis pathway [1,2,3]. The prevalence of the genetic defect is considered high (~1 in 1700 individuals), although the clinical penetrance of the disease is very low (≤1%) [4]. The main clinical manifestation of AIP is acute neurovisceral attacks characterized by abdominal pain, often accompanied by severe fatigue, nausea, vomiting, constipation, and appetite loss. Patients may also have hypertension, tachycardia, trouble sleeping, and anxiety. Severe attacks can lead to hyponatremia, seizures, sensory loss, or motor neuropathy [5].

Acute attacks are associated with the high accumulation of porphyrin precursors, δ-aminolevulinic acid (ALA) and porphobilinogen (PBG), when hepatic heme synthesis is up-regulated by endogenous or exogenous factors, such as fasting, hormonal fluctuations during the menstrual cycle, infection, stress, smoking, alcohol, or exposure to porphyrinogenic drugs. All these factors strongly induce the transcription of the first and rate-limiting enzyme in the heme synthesis pathway, *ALA Synthase 1* (ALAS1, EC 2.3.1.37) in hepatocytes [1,6,7,8]. Substances such as endogenous intermediates and xenobiotics, metabolized by the CYP450 enzyme system in the liver, can result in the enhanced transcription of both specific CYP450 and *ALAS1* through nuclear receptors binding a cis-acting regulatory drug-responsive sequence (ALAS drug-responsive element, ADRES) located upstream of the promoter of such genes [9]. Fasting, acting through the nuclear receptor peroxisome proliferator-activated receptor-alpha (PPARα) and the PPAR-gamma coactivator-1 alpha (PGC-1α), can also induce a direct transcriptional up-regulation of hepatic *ALAS1* [10,11]. A third potential mechanism of hepatic *ALAS1* up-regulation is associated with the inducible and highly dynamic heme oxygenase-1 (HMOX1, EC 1.14.14.18), which produces an active hepatic heme turnover in response to cell stress or inflammatory and infectious diseases [12]. Finally, the exacerbated abundance of intracellular heme decreases the stability of ALAS1 mRNA, inhibits the uptake of pre-ALAS into mitochondria, and reduces the stability of the mitochondrial ALAS1 protein via Lon Peptidase 1 (LONP1)-mediated degradation [13].

The first-line therapeutic approach for acute attacks is based on both carbohydrate loading and intravenous hemin therapy to down-regulate hepatic *ALAS1* transcription. Hemin replenishes the regulatory heme pool in hepatocytes and is more effective than glucose [7,14]. Carbohydrate loading (300 to 500 g/day), based on oral or intravenous glucose infusions, has been used to alleviate relapses or milder attacks with low requirements for narcotics and the absence of hyponatremia or motor impairment [6,7]. However, recent studies have not shown a clear preventive effect. While high carbohydrate intake and the subsequent increase of insulin levels were associated with lower biochemical disease activity in a case-control trial performed in northern Norway [15], prophylactic infusion of dextrose or a carbohydrate-rich diet have yielded inconclusive clinical findings in epidemiological studies in the USA [16].

Early studies reported an abnormal oral glucose tolerance test (GTT) and hyperinsulinemia in patients with AIP [17,18], which resemble the findings in cellular insulin resistance (IR). More recently, we have reported a delayed GTT, serum hyperinsulinemia, and abnormal carbohydrate metabolism in AIP mice [19]. Since insulin promotes hepatic *ALAS1* transcription repression through the PI3-K/Akt system [10], IR could reduce the efficiency of carbohydrate loading as a treatment. Thus, the first aim of this study was to evaluate the prevalence of IR in patients with AIP in an observational case-control study. In AIP mice, we assayed the synergistic effect of glucose and two types of insulin (a fast-acting and an experimental liver-targeted insulin [20] as a treatment and prevention therapy for acute attacks induced both by fasting and by challenging the mice with multiple increasing doses of a porphyrinogenic drug. The novel liver-targeted insulin is a fusion protein formed by the insulin B chain followed by a linker QRGGGGGQR [21], the insulin A chain, the short linker GAP, and the apolipoprotein A-I. This fusion protein prolongs the insulin half-life in circulation and increases insulin activity in the liver. In the db/db model of metabolic syndrome, subcutaneous administration of the fusion protein reduced body weight and improved steatosis [20]. In this study, the activity of this novel liver-targeted insulin is evaluated in a mouse model of AIP.

## 2. Materials and Methods

### 2.1. Reagents

Fast-acting insulin (Actrapid^®^) was from Novo Nordisk Production SAS, Chartres, France. The recombinant fusion protein insulin fused to apolipoprotein A-I (Ins-ApoAI) and apolipoprotein A-I (Apo) were expressed in *E. coli* and purified by GenScript Corp. (Piscataway, NJ, USA).

### 2.2. Participants and Study Design

A case-control study was conducted in 44 patients with AIP and 55 age- and gender-matched healthy volunteers. Participants were recruited during the follow-up in the Porphyria Unit at the Hospital Universitario 12 de Octubre from May 2018 to May 2019. Among patients, there was high variability in the *PBGD* gene mutation, with the most prevalent being the 340-341insT (4 of 44 cases). Control volunteers were selected among family members of the AIP patients once the presence of the family mutation and biochemistry compatible with acute porphyria had been ruled out. The study was approved by the Hospital Ethics committee (CEIm: 19/262). A medical doctor questioned participants about the presence or absence of AIP symptoms, the time of diagnosis, the number and duration of attacks, and about triggering and relieving factors during attacks. Blood sampling was routinely performed between 08:00 and 10:00 a.m. after overnight fasting, according to hospital protocols. The data collected included a physical examination and laboratory tests (glucose, insulin, and urinary ALA and PBG levels). Factors related to the development of insulin resistance were also collected: (i) metabolic syndrome [22], (ii) overweight [23] measured according to the WHO recommendations, (iii) sedentary lifestyle [24] according to the international physical activity questionary (IPAQ, www.ipaq.ki.se., last entry March 3, 2021), (iv) the presence of polycystic ovary syndrome (PCOS) [25], (v) glucocorticoid treatment [26], (vi) human immunodeficiency virus infection [27], and (vii) genetic syndromes of severe IR [28]. Hypertension (HT) was defined as a blood pressure ≥130/≥80 mmHg, HOMA was calculated as described by Matthews et al. [29], and insulin resistance was defined as a HOMA-IR index of >3.4, corresponding to the 90th percentile of the HOMA-IR index distribution in a Spanish adult nondiabetic population [30]. Sedentary lifestyle was measured according to the international physical activity questionary (IPAQ) and dichotomized into sedentary (category 1) or non-sedentary lifestyle (categories 2 and 3). Metabolic syndrome was defined according to the American College of Endocrinology criteria (among other parameters BMI ≥ 25 kg/m^2^ or waist circumference ≥102 cm [men] or ≥88 cm [women] and fasting glucose ≥6.1 mmol/L (≥110 mg/dL) [22]).

### 2.3. Experimental Studies in a Murine Model of AIP 

AIP mice (C57BL/6-^pbgdtm1(neo)Uam^/C57BL/6-^pbgdtm2(neo)Uam^) exhibit hepatic PBGD activity reduced to 30% of normal and reproduce the biochemical test results, together with the presence of pain and motor neuropathy that characterize human porphyria [31]. Experimental protocols were approved by the Ethics Committee of the University of Navarra (CEEA032-13) according to European Council guidelines.

The glucose tolerance test (GTT) was performed after 15 h of fasting. Male mice were intraperitoneally administered with one dose of 20% glucose (10 μL/g mice). Glycemia was quantified 5 min later, followed by one single subcutaneous administration of Ins-ApoAI (90, 30, or 10 µg/kg; dose eq. to 2.3, 0.77 or 0.26 μg of pure insulin/mouse) or fast-acting insulin (180 µg/kg or 18 µg/kg; dose eq. to 4.55 or 0.45 μg of pure insulin/mouse). Glycemia was quantified every 30 min using the Accu-Chek^®^ Aviva meter (Roche, Sant Cugat del Vallès, Spain) for 6 h. Supplementary doses of glucose were administered when serum glucose levels were lower than 100 mg/dl. After 6 h, the animals were euthanised and liver samples were frozen at −80 °C. 

The steady-state mRNA levels of the genes were analyzed by quantitative RT-PCR using iQ SYBR Green supermix in an iQ5 real-time PCR detection system (Bio-Rad, Hercules, CA, USA) and specific primers (*alas1*, forward: 5′-CAAAGAAACCCCTCCAGCCAATGA-3′, reverse: 5′-GCTGTGTGCCGTCTGGAGTCTGTG-3′, product length: 104 bp; *pgc-1α*, forward: 5′-GAAGTGGTGTAGCGACCAATC-3′, reverse: 5′-AATGAGGGCAATCCGTCTTCA-3′, product length: 162 bp; *hmox1*, forward: 5′-CCAGAGTGTTCATTCGAGCA-3′, reverse: 5′-CTGCAGGGGCAGTATCTTGC-3′, product length: 116 bp; *g6pase*, forward 5′-AACGCCTTCTATGTCCTCTTT-3′, reverse: 5′-GTTGCTGTAGTAGCTGGTGTC-3′, product length: 168 bp; *pepck*, forward: 5′-AGCCTGCCCCAGGCAGTGAG-3′, reverse: 5′-CATGCACCCTGGGAACCTGGC-3′, product length: 339 bp and *cyp7a1*, forward: 5′-GCTGTGGTAGTGAGCTGTTGCA-3′, reverse: 5′-CACAGCCCAGGTATGGAATCA-3′, product length: 103 bp). PCR amplification was performed under the following conditions: one cycle of 3 min at 95 °C, followed by 35 cycles of 15 s at 95 °C, 30 s at 60 °C, 30 s at 72 °C, and 30 s at the detection temperature of each gen, followed by a single final extension cycle of 72 °C for 4 min. The amount of gene transcript was calculated as the n-fold difference relative to the control gene *actin* (forward: 5′-CGCGTCCACCCGCGAG-3′, reverse: 5′-CCTGGTGCCTAGGGCG-3′, product length: 125 bp). The results were expressed according to the formula 2^Ct(Actin)-Ct(gene)^, where Ct represents the difference in threshold cycle between the target and control genes.

The treatment after the co-administration of glucose and exogenous insulin was studied during an ongoing acute attack in male AIP mice. Acute attack was induced by an intraperitoneal administration of four increasing doses of phenobarbital (75, 80, 85, and 90 mg/kg) at 24 h intervals. On day 3 and 4, mice were treated with glucose one hour before and nine hours after the administration of phenobarbital. Co-administration with insulin was performed 5 min after the first daily dose of glucose (18 µg/kg of fast-acting insulin or 90 µg/kg of Ins-ApoAI). Mice were housed in metabolic cages (BIOSIS Biologic Systems, SL, Madrid, Spain), and urine samples were collected for 24 h. On day 5, animals were euthanised 20 min after an additional administration of 90 mg/kg of phenobarbital, and then liver tissue samples were frozen at −80 °C.

In a prevention trial, male mice were fed *ad libitum* and two glucose doses (2 h apart) were injected daily between days −5 and +4. Animals received Ins-ApoAI on days –5 and 0, two hours before the first dose of phenobarbital. Co-administration with Ins-ApoAI (90 µg/kg, sc) was performed two hours before the first dose of phenobarbital. Pain scores and motor coordination were measured four hours after the fourth dose of phenobarbital injection, as previously described [32]. After each dose of phenobarbital, mice were housed in metabolic cages, and urine samples were collected after 24 h. Urinary excretion of ALA and PBG were quantified using a quantitative ion exchange column method (BioSystems SA, Barcelona, Spain) and measured at 555nm in an Ultrospc 3000 spectrophotometry (Pharmacia Biotech, Buckinghamshire, UK).

The ratio of mitochondria per hepatocyte was scored in different groups of male mice between 8 and 10 weeks old after a 10-day protocol of glucose administration combined or not with fast-acting insulin (10 ui/mL, eq. to 18 µg/kg of crystallized insulin equivalent), 1 mg/kg of ApoAI or 1 mg/kg of Ins-ApoAI (equivalent to 90 µg/kg of crystallized insulin) on days 1, 4, 6, and 8. Animals were euthanised on day 11. An immunohistochemistry assay was performed on liver tissue samples using an antibody to detect mitochondria MTCO1 protein, 1:4000 dilution (ref: 1D6E1A8, ab14705 abcam, Cambridge, UK). Detection and counting of mitochondria in histological images was carried out using a plugin developed for Fiji/ImageJ, an open-source Java-based image processing software [33]. The plugin was developed by the Imaging Platform of the Cima Universidad de Navarra. The image processing pipeline includes automatic tissue detection, individual color-channel retrieval (hematoxylin and DAB) using a color deconvolution plugin for stain separation [34], and automatic nuclei detection and counting from the hematoxylin channel and mitochondria detection and counting from the DAB channel. The proportion of anti-MTCO1 stained mitochondria was counted from at least 1200 nuclei from four microscopic fields from each liver.

The body composition was measured in 15-h-fasted mice by quantitative magnetic resonance (QMR) technology (EchoMRI-100-700, Echo Medical Systems, Houston, TX, USA) as previously described [35]. At the end of the experimental period, mice were euthanised, and blood and tissue samples including liver, kidney, heart, soleus and gastrocnemius muscles, white adipose tissue (WAT) (gonadal, retroperitoneal, mesenteric, and subcutaneous), and brown adipose tissue depots (BAT) were collected as previously described [36]. The visceral WAT was estimated by the sum of gonadal, retroperitoneal, and mesenteric depot weights.

### 2.4. Statistics 

The results were plotted as the mean ± s.d. The Fisher’s exact test was used to compare the distribution of qualitative data. Prior to statistical analysis, quantitative data were transformed using the formula Log (1 + x) in order to normalize the variances. Comparisons between two groups were analyzed by Student’s *t* test. In the case of comparisons across more than two groups, data were analyzed with the ANOVA test, and pairwise comparisons were made using Bonferroni´s multiple comparison tests. The null hypothesis was rejected when *p* < 0.05. Statistical analysis was performed using GraphPad Prism^®^ 5 (GraphPad Software, Inc., La Jolla, CA, USA) 

## 3. Results

### 3.1. High Prevalence of a Pathological HOMA-IR Index in Patients with AIP

**Figure 1:**
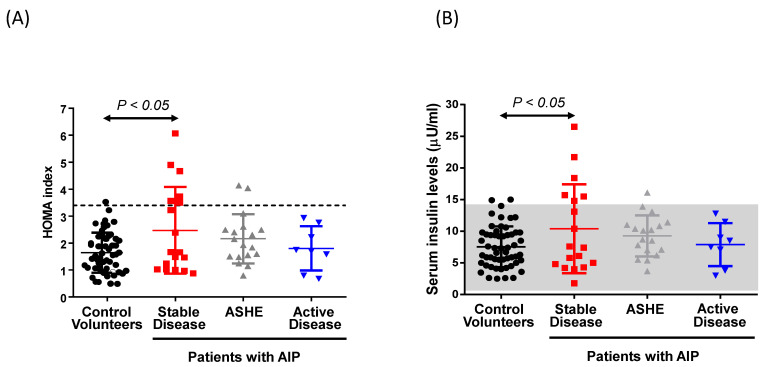
Range distribution of the HOMA-IR index and serum insulin levels in control volunteers and carriers of a mutation in the *PBGD* gene classified according to biochemical and clinical characteristics in patients with stable disease (SD), asymptomatic high excreters (ASHE), or those with active disease (AD). (**A**) The HOMA-IR index and (**B**) serum insulin levels significantly increased in patients with stable disease (AIP-SD). Dotted lines indicate the cut-off values of the HOMA-IR index (≥3.4) and the gray rectangle corresponds to the 95% confidence interval of serum insulin levels corresponding to the group of voluntary controls. Comparisons were performed by one-way-ANOVA followed by Bonferroni post-hoc correction.

Forty-four Spanish patients with AIP and 55 control volunteers (CV) matched by age, gender, and BMI were enrolled in an observational case-control study in order to estimate the homeostasis model assessment of insulin resistance (HOMA-IR) index. Subjects had no HIV infection, genetic syndromes associated with severe IR, or steroid treatment. Clinical and biochemical descriptions of cases and control volunteers are shown in Table 1. A significantly higher frequency of pathological levels of the HOMA-IR index (cut-off ≥ 3.4) [30] was observed in eight (18.2%) patients with AIP as compared to one single subject (1.82%) in the CV group (*p* = 0.01, Fisher’s exact test) (Figure 1a). The only individual included in the CV group showing a high HOMA-IR index was also overweight and met the criteria for metabolic syndrome (see Material and Methods). Among the eight AIP cases, a high HOMA-IR index was associated with obesity (BMI ≥ 30) [37] in five participants; one had polycystic ovary syndrome (associated with insulin resistance as reported in [25]), and two showed no specific etiology related to IR other than porphyria. Finally, a sedentary lifestyle was found to be more frequent among CV (45%) than in patients with AIP (21%) (Table 1).

Among the 44 individuals carrying a mutation in the *PBGD* gene, eight (18%) were classified with active disease (AIP-AD) as defined by high urinary porphyrin precursor excretion (ALA, ≥7.5 mmol/mol creat.; PBG, ≥3.4 mmol/mol creat.) and at least one attack in the last year or were under prophylactic hemin treatment (Figure 1a). Eighteen cases (41%) showed high urinary precursor levels but clinically stable disease and these constituted the asymptomatic high excreters (AIP-ASHE) group. Furthermore, 18 individuals (41%) with clinically stable disease and low urinary precursor excretion (ALA, <7.5 mmol/mol creat.; PBG, <3.4 mmol/mol creat.) were included in the stable disease (AIP-SD) group. Of interest, among patients with high HOMA-IR (Figure 1a) and hyperinsulinemia (Figure 1b), six were classified as AIP-SD (33% of the group) and two were AIP-ASHE (11%), whereas none of the AIP-AD group showed values outside the normal range for this index. Indeed, only the HOMA-IR index (Figure 1a) and serum insulin levels (Figure 1b) from the AIP-SD group showed significant differences with the CV group. These data suggest that increased serum insulin levels are associated with a biochemical and clinical improvement in patients with AIP.

### 3.2. Ins-ApoAI Induced a Fast and Sustained Normalization in the Gene Transcription Involved in the Liver Regulation of Heme Synthesis, Gluconeogenesis, and Bile Acid Synthesis in Fasted WT and AIP Mice

**Figure 2:**
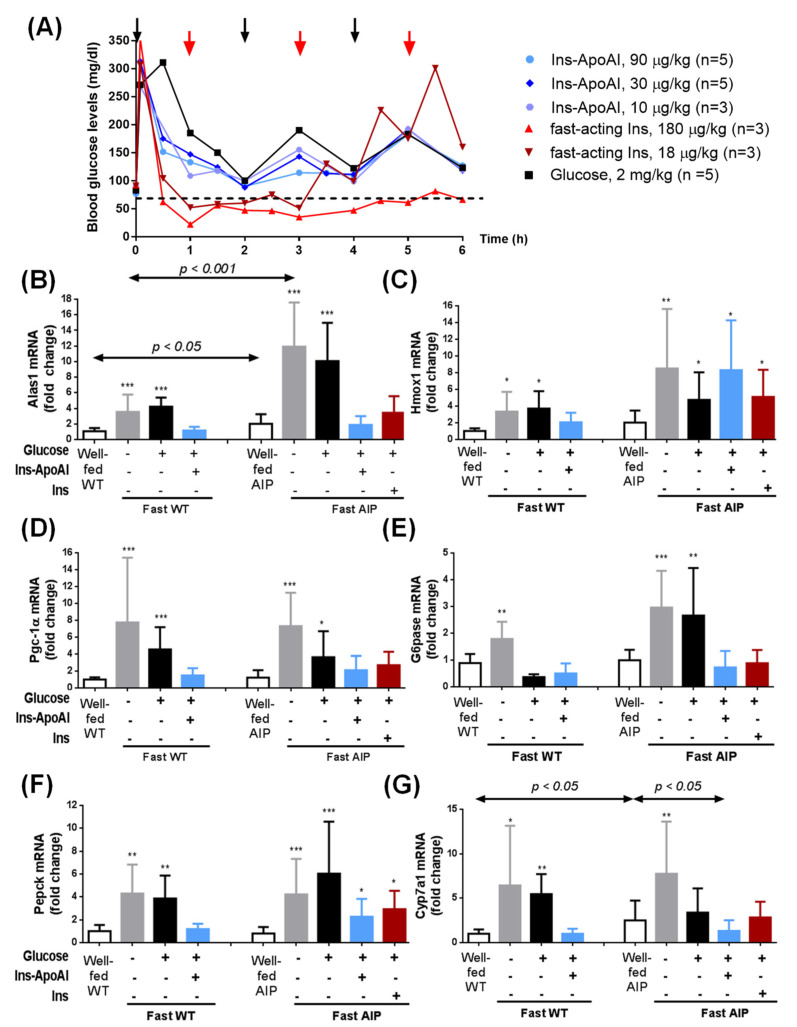
Transcriptional analysis of important genes in fasted animals treated with glucose, and glucose with a fast-acting insulin or an experimental liver-targeted insulin (Ins-ApoAI). (**A**) Serum glucose kinetics over 6 h measured after glucose overloads in 15-h-fasted AIP mice treated with glucose. Glycemia was measured at 30 min intervals, starting 5 min post-initial dose. Black arrows represent glucose administration for all groups and red arrows represent supplementary glucose administration for the fast-acting insulin group. Kinetics of the (**B**) *alas1*, (**C**) *hmox1*, (**D**) *pgc-1α*, (**E**) *g6pase*, (**F**) *pepck*, and (**G**) *cyp1a7* gene transcription in the liver were measured in male WT and AIP mice at baseline (well-fed condition), 15-h-post starvation and after the administration of three doses of glucose (2 mg/kg, i.p.), three doses of glucose with a single subcutaneous dose of Ins-ApoAI (eq. from 90 µg/kg of crystallized insulin equivalent) or six doses of glucose with a single dose of a commercial fast-acting insulin (10 ui/mL, eq. to 18 µg/kg). Data are mean ± s.d. of five animals per group. Comparisons were performed by one-way ANOVA followed by Bonferroni post-hoc correction. Alas1, aminolevulinate synthase 1; hmox1, heme oxygenase-1; pgc-1α, Peroxisome proliferator-activated receptor-gamma coactivator-1 alpha; g6pase, glucose 6-phosphatase; pepck, phosphoenolpyruvate carboxykinase and cyp1a7, cholesterol 7 alpha-hydroxylase. *, *p* < 0.05; **, *p* < 0.01; ***, *p* < 0.001 vs. well-fed mice and. Ins-ApoAI, the fusion protein of a single chain insulin and apolipoprotein A-I.

The transcriptional effects of the exogenous administration of glucose and/or insulin on important genes related to hepatic heme synthesis were evaluated in fasted mice injected with both fast-acting insulin (Actrapid^®^) and an experimental liver-targeted insulin, Ins-ApoAI. The matched glucose/insulin doses were titrated with a GTT in 15-h-fasted AIP mice (Figure 2a). The mice received three doses of 2 mg/kg of glucose (20% solution) every 2 h throughout the 6 h of study and supplementary doses when they were close to hypoglycemic values (~70 mg/dL). Co-administration with Ins-ApoAI or fast-acting insulin was performed 5 min after the first glucose dose. Mice treated with subcutaneous Ins-ApoAI (0.11, 0.33 and 1 mg/kg of Ins-ApoAI, equivalent to 10, 30, and 90 µg/kg of crystallized insulin equivalent, respectively) showed blood glucose kinetics similar to the control group (Figure 2a) and received a total of three doses of glucose. In contrast, mice that received fast-acting insulin (10 and 100 ui/mL, equivalent to 18 µg/kg and 180 µg/kg of crystallized insulin) showed a rapid organ glucose uptake and needed up to six doses of glucose (one dose/h). At the end of the study, the administration of three doses of glucose alone did not reduce hepatic *alas1* over-expression, but the administration of the highest doses of Ins-ApoAI (30 and 90 µg/kg) and the lowest fast-acting insulin (18 µg/kg) reduced its expression to values found in fed AIP mice (Supplementary Figure S1). The doses with the best outcomes (i.e., 90 µg/kg Ins-ApoAI and 18 µg/kg fast-acting insulin) were chosen for further transcription studies of the genes of interest in AIP and WT mice.

The transcription of genes involved in the regulation of hepatic heme synthesis (*alas1, pgc-1α*) and catabolism (*hmox1*) were strongly induced in both male WT and AIP mice after a 15-h-fasting period (Figure 2b–d). Of interest, the fold-change expression for *alas1* was significantly higher in AIP than in WT animals, both in the fed (2-fold induction vs. WT mice) and fasted conditions (3.4-fold induction vs. WT mice) (Figure 2b). Gluconeogenesis, as measured by the fold-change up-regulation of *glucose 6-phosphatase* (*g6pase*) (Figure 2e) and *phosphoenolpyruvate carboxykinase* (*pepck*) (Figure 2f) genes, was also significantly activated in fasted WT (2-fold and 4.3-fold induction, respectively) and AIP mice (3-fold and 5.3-fold induction, respectively). 

The expression of genes involved in the heme synthesis pathway, *alas1*, and the *pgc1α* gene, returned to normal after co-administration of glucose with the exogenous insulins in both WT and AIP mice (Figure 2b,d) but not with glucose alone. To better understand what occurs in the regulation of hepatic *alas1*, a group of mice was euthanised 2.5 h after the end of fasting. Alas-1 tended to return to baseline levels 30 min after the second glucose administration (Supplementary Figure S2a). However, 6 h after ending the fast (2 h after the third glucose dose) its expression showed a re-induction to near fasting values while both *alas1* (Figure 2b and Supplementary Figure S2a) and *pgc-1α* (Figure 2c) gene transcription displayed pre-fasting levels in both insulin-treated groups. Regarding protein analysis, we detected a low ratio of pAkt/Akt in the liver of 15-h-fasted mice when compared with animals fed ad libitum (Supplementary Figure S3). The co-administration of glucose and Ins-ApoAI significantly increased the pAkt/Akt ratio within 10 min, whereas this was delayed until 2.5 h in mice treated with glucose alone. Based on these results, it is tempting to speculate that pAkt induces rapid Forkhead Box O1 (FOXO1) phosphorylation and breaks up the FOXO1/PGC1α complex, causing the sustained repression of alas1 transcription in the liver of AIP mice treated with Ins-ApoAI.

Another difference between WT and AIP mice was the down-regulation of the key limiting enzyme of the hepatic heme catabolism. Although, fasting induced the expression of *hmox1* in both strains (3.3-fold induction in WT and 4.2-fold induction in AIP mice), co-administration of exogenous insulin treatment only normalized its expression in WT mice (Figure 2c).

In WT mice, the administration of three doses of glucose normalized the expression of *g6pase*, whereas in AIP mice its expression was only normalized after co-administration of glucose with the exogenous insulins (Figure 2e). *Pepck* up-regulation was normalized after co-administration with glucose and Ins-ApoAI in fasted WT mice, whereas in the liver of AIP mice it remained overexpressed when compared to pre-fasted values (Figure 2f). These data suggest a differential regulation of the glucose supply from the liver to the bloodstream during fasting in the livers of WT and AIP mice. Treatment with glucose overload tended to normalize the expression of *g6pase* and *pepck* at 2.5 h (30 min after the second dose of glucose), but their expression increased again at 6 h, that is, 2 h after the third dose of glucose (Supplementary Figure S2b,c). Co-administration of glucose and Ins-ApoAI or fast-acting insulin induced better transcriptional control of both gluconeogenic enzymes throughout the study period (Supplementary Figure S2b,c); however, fast-acting insulin required double the dose of glucose to maintain the same transcriptional level of these two enzymes when compared to Ins-ApoAI.

Finally, transcription levels of the *cyp7a1* gene, an insulin-regulated gene encoding the enzyme cholesterol 7α-hydroxylase (EC 1.14.14.23) which catalyzes the initial step in bile acid synthesis [38] also showed a significant induction in fasted mice (Figure 2g). Well-fed AIP mice showed a significant overexpression of the insulin-dependent *cyp7a1* gene compared to WT mice (2.7-fold induction), and fasting strongly induced its over-expression in both strains (6.4-fold induction in WT and 2.9-fold induction in AIP mice) (Figure 2g). Of interest, the administration of glucose and glucose with rapid insulin reduced this up-regulation to values within the range observed in fed AIP mice while co-administration of glucose and Ins-ApoA1 normalized its hepatic expression when compared to well-fed WT mice (Figure 2g). These data suggest that *cyp7a1* regulation could be affected by a hepatocyte insulin resistance in AIP mice that is reversed with the administration of Ins-ApoAI.

### 3.3. The Relative Contribution of Insulin to Protect against alas1 Induction Modulated by Barbiturate Challenge

**Figure 3:**
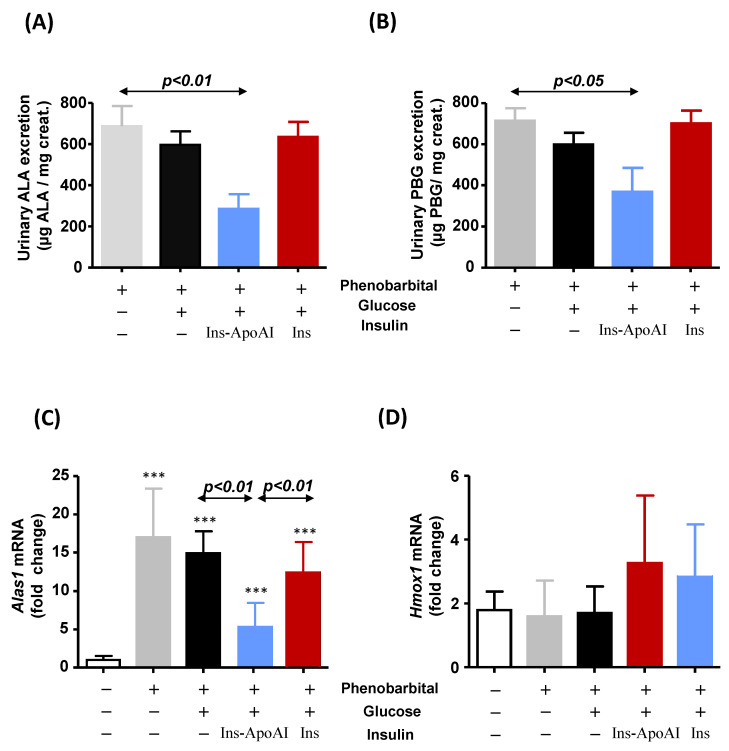
Therapeutic efficacy of subcutaneous administration of Ins-ApoAI and glucose during an ongoing phenobarbital-induced attack. Urinary (**A**) ALA and (**B**) PBG excretion on day 4 of the phenobarbital-induced attack. While co-administration of glucose and Ins-ApoAI halved the excretion, glucose alone or glucose co-administered with fast acting insulin failed to reduce the excretion of the neurotoxic precursors ALA and PBG. Hepatic expression of (**C**) *alas1* and (**D**) *hmox1* measured on day 5, 20 min after a supplementary phenobarbital dose of 90 mg/kg. Data are mean ± s.d. of at least four animals per group. Comparisons were performed by one-way ANOVA followed by Bonferroni post-test. ***, *p* < 0.001 vs. control untreated AIP mice. Daily urinary excretion of porphyrin precursors corresponding to baseline values are: 52 ± 12.7 µg ALA/mg creat. and 11.8 ± 4.2 µg PBG/mg creat. *Alas1, aminolevulinate synthase 1; hmox1, heme oxygenase-1*. Ins-ApoAI, the fusion protein of a single chain insulin and apolipoprotein A-I.

Drugs up-regulate the *alas1* gene via direct interactions with 5’-upstream response elements in the promoter region of the gene. Given that it is an enhanced transcriptional pathway different from that activated during fasting, we decided to test the co-administration of glucose and exogenous insulin during acute attacks of porphyria induced by the administration of increasing doses of phenobarbital for four consecutive days. In a treatment study, the administration of glucose or glucose and fast-acting insulin did not alter daily ALA (Figure 3a) and PBG (Figure 3b) excretion on the last day of the study (24 h after the fourth-last dose of phenobarbital). However, the administration of glucose and Ins-ApoAI halved the excretion of both precursors. On day five, mice received a supplementary dose of phenobarbital (90 mg/kg) and were euthanized 20 min later to study the hepatic expression of the genes involved in the heme synthesis and catabolism. While glucose administration with or without fast-acting insulin did not repress *alas1* transcription, co-administration of Ins-ApoAI reduced its overexpression by 60% (Figure 3c). Insulin, either free or conjugated with ApoAI, tended to induce the expression of *hmox1*, although no significant differences were observed between groups (Figure 3d).

**Figure 4:**
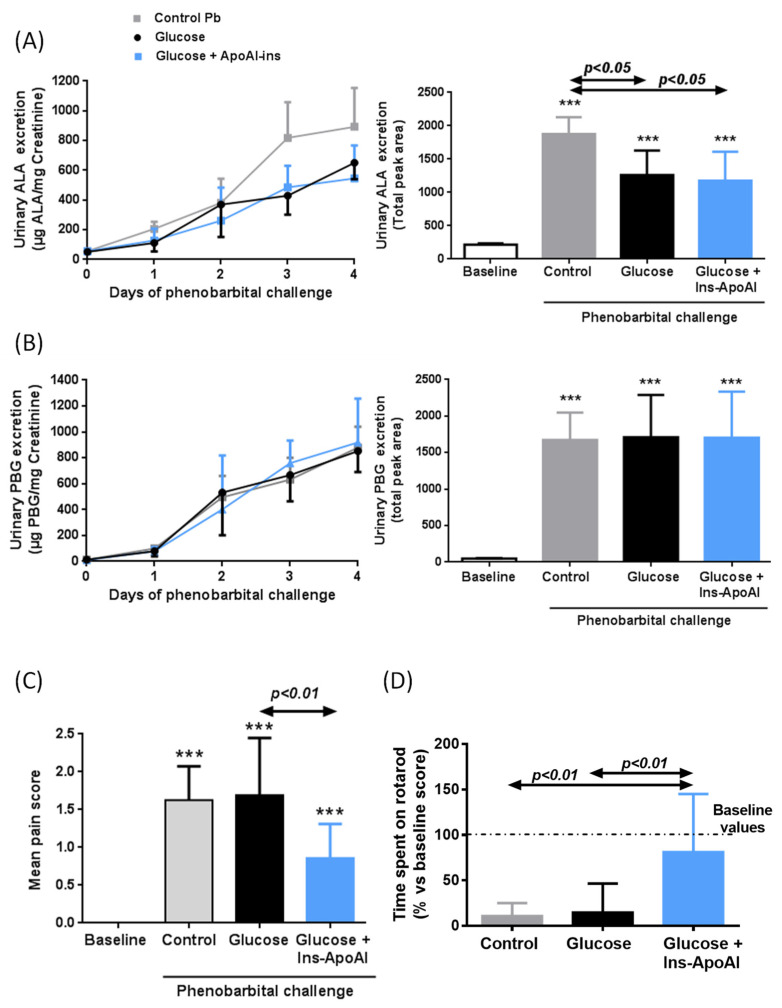
Therapeutic efficacy of multi-dose co-administration of glucose and liver-targeted insulin against a drug-induced attack in AIP mice. Daily urinary (**A**) ALA and (**B**) PBG excretion (left) and quantification of the area under the curve over time (right) during a prevention trial (as detailed in material and method section). (**C**) Pain scoring measured by the mouse grimace scale (MGS) and (**D**) motor coordination score assessed by the rotarod test (% respect baseline score) performed 4 h after the fourth dose of phenobarbital. Data are mean ± s.d. of six animals per group. Comparisons were performed by one-way ANOVA followed by Bonferroni post-test. ***, *p* < 0.001 vs. baseline values. Glu, Glucose; Ins-ApoAI, the fusion protein of a single chain insulin and apolipoprotein A-I.

In a prevention study, the protective effect of the recurrent administration of Ins-ApoAI and/or glucose was assayed in AIP mice to mimic the prophylactic therapy some patients receive when beginning with prodromal symptoms associated with an acute attack (Figure 4). While PBG levels were unchanged (Figure 4b), urinary ALA quantification measured as daily excretion during the four days of phenobarbital challenge (Figure 4a, left) or area under the curve (Figure 4a, right) showed a significant reduction in the two groups treated with glucose, with or without Ins-ApoAI (62 or 67% vs. the control phenobarbital group). However, this reduction was not translated into a clinical improvement in phenobarbital–challenged AIP mice treated with glucose alone, as measured by both a pain score (Figure 4c) and motor coordination in the rotarod test (Figure 4d). Of interest, these two parameters showed a significant improvement in the group that received Ins-ApoAI together with glucose, as compared with mice treated with glucose alone (Figure 4c,d).

**Figure 5:**
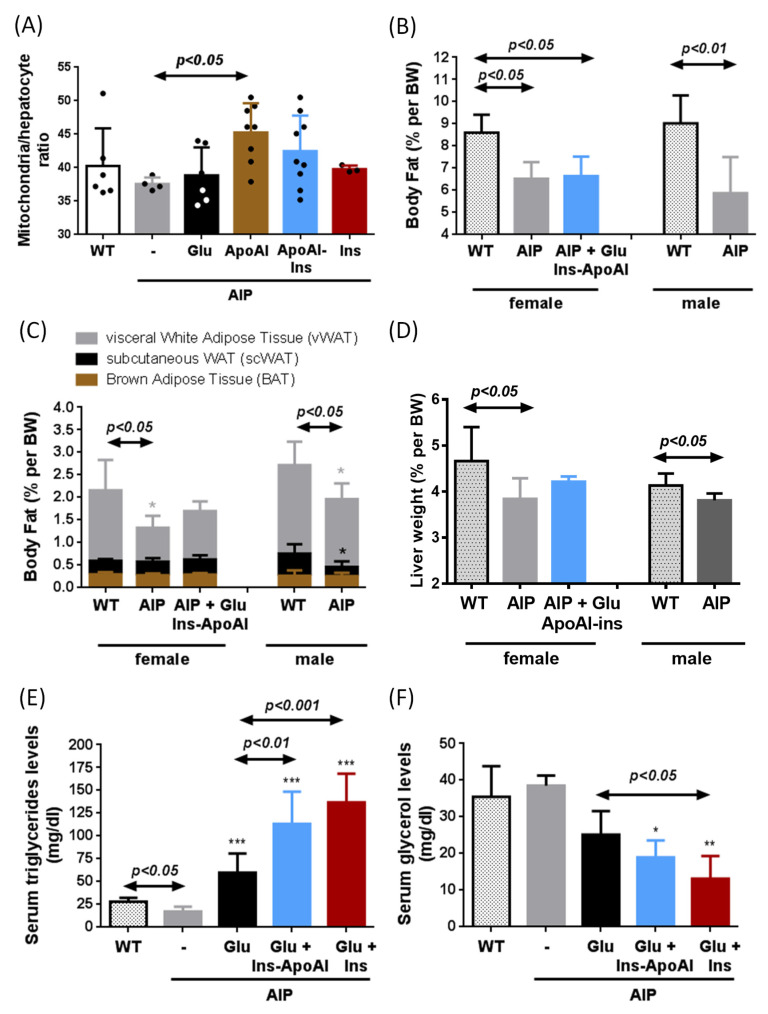
Changes in mitochondrial count per hepatocyte, serum levels of triglycerides and glycerol and body mass composition of WT and AIP mice after repeated administration of glucose and exogenous insulin. (**A**) The ratio of mitochondria per hepatocyte was measured in AIP mice treated with glucose combined or not with fast-acting insulin, ApoAI or Ins-ApoAI. (**B**) Body fat composition, (**C**) brown adipose tissue (BAT), visceral white adipose tissue (vWAT), and subcutaneous white adipose tissue (scWAT) measured in fasted mice after 15 h of starvation using quantitative magnetic resonance. (**D**) Liver weight in WT and AIP mice. Serum (**E**) triglyceride and (**F**) glycerol levels measured in male mice treated with glucose for 10 days combined or not with four doses of either fast-acting insulin or Ins-ApoAI. All the assays presented in this figure were performed in mice not challenged with phenobarbital to avoid interference from the barbiturate effect. WT: wild type; AIP: acute intermittent porphyria; Glu: glucose; Ins: fast-acting insulin; Ins-ApoAI: Apolipoprotein AI conjugated with insulin. Data are mean ± s.d. of four mice per group. Comparisons were performed by one-way ANOVA followed by Bonferroni post-test. *, *p* < 0.05; **, *p* < 0.01; ***, *p* < 0.001 vs. control untreated AIP mice. Glu: glucose; Ins-ApoAI: the fusion protein of a single chain insulin and apolipoprotein A-I; Ins: fast-acting insulin.

An important finding observed in the liver of AIP mice treated with repeated doses of Ins-ApoAI was an increase in the number of mitochondria per hepatocyte (Supplementary Figure S4). We suggest that an increase in the mitochondria per hepatocyte ratio together with the glucose supplement could enhance energy synthesis. In order to better explore the effect of ApoAI, new cohorts of AIP mice were exposed to the 10d-protocol with glucose with or without exogenous insulin. These animals were not challenged with phenobarbital to avoid interference from the barbiturate effect. Of interest, the administration of ApoAI significantly increased the number of mitochondria per hepatocyte (120% vs. control AIP) (Figure 5a). Mice injected with Ins-ApoAI also showed an increased ratio (113% vs. control AIP), although differences were not statistically significant due to high intragroup dispersion. No changes were observed in the liver of mice co-injected with glucose and fast-acting insulin (Figure 5a).

Regarding body composition during starvation, AIP mice showed reduced fat accumulation when compared to age-matched WT mice, suggesting a predominance of the lipid catabolic processes in white adipose tissue (WAT) of AIP mice (Figure 5b). AIP mice showed reduced fat mass in both females (75% vs. WT group) and males (65% vs WT group) (Figure 5b). Within the fat tissue, a significant reduction in the percentage of visceral WAT (vWAT) depots was observed in AIP mice, with no changes in brown adipose tissue (BAT) (Figure 5c). In female AIP mice, a 10 day-protocol of glucose (two doses of 2 mg/kg, 2 h apart) together with four doses of Ins-ApoAI (every three days) did not modify the weight of vWAt (Figure 5c), or the weight of other organs such as the liver (Figure 5d), heart, kidney, or the soleus and gastrocnemius muscles (data not shown). 

Fasted AIP mice showed low levels of serum triglycerides (TG, 61% as compared to WT mice) (Figure 5e). TG levels (Figure 5e) were increased in AIP mice treated with glucose (3.4-fold increase) (two doses of 2 mg/kg, 2 h apart) for 10 days and serum levels rose even higher after co-administration with exogenous insulin, both fast-acting insulin (7.8-fold increase) or Ins-ApoAI (6.5-fold increase) (four doses, three days apart). These data suggest that the co-administration of glucose and insulin increases the availability of circulating high-energy molecules in AIP mice. While serum TG levels rose, serum glycerol levels were significantly reduced in insulin-treated animals (Figure 5c), according to the lipogenic/antilipolytic effect previously described during insulin treatment [39,40].

## 4. Discussion

Carbohydrate loading is a well-documented treatment for mild acute attacks of porphyria [7,8]. Fasting and glucagon up-regulate the hepatic ALAS1 gene by the interaction of the complexes formed by PGC-1α with the FOXO1 and nuclear respiratory factor 1 (NRF-1) with a sequence within the ALAS1 promoter. The therapeutic effect of carbohydrates is based on their ability to induce endogenous insulin synthesis and disrupt the FOXO1-PGC1α complex through the hepatic pAkt signaling [10]. However, early reports showed abnormal oral glucose tolerance tests, as well as hyperinsulinemia in patients with AIP [17,18,41,42] which could lead to the reduced effectiveness of the glucose-treatment. The nature of IR in patients with AIP remains unknown. Chronic ALA accumulation, as occurs in patients with active disease, can increase reactive oxygen species (ROS), which could cause the dysfunction of glutathione-insulin transhydrogenase (the enzyme responsible for insulin degradation) [43]. However, the effect of ALA accumulation is ruled out in our study because patients with active disease (the AIP-AD group) showed serum insulin levels and a HOMA-IR index within the normal range. Of interest, *PBGD* mutation carriers with hyperinsulinemia report no clinical symptoms related to AIP. Similarly, Storjord et al. also found higher serum insulin levels associated with lower biochemical disease activity in their patient cohort [15]. These data suggest that IR and high-serum insulin levels are not a consequence of disease activity, but rather that sustained hyperinsulinemia can protect against acute attacks of porphyria.

Here, we assayed the efficacy of repressing *alas1* transcription after the administration of a commercial fast-acting insulin or an experimental hepatotropic Ins-ApoAI in an AIP mouse model. In these mice, abnormal GTT, hyperinsulinemia, and blockage of glycogenolysis pathways have been previously reported [19,44]. Significant repression of the hepatic *alas1* and *pgc1α* gene expressions were observed 6 h post-insulin administration with both fast-acting and Ins-ApoAI in fasted AIP mice, while treatment based exclusively on glucose did not yield a comparable decrease. Our data show that the administration of exogenous insulin quickly increased the pAkt/Akt ratio and induced a sustained repression of hepatic alas1 transcription, through the breakup of the transactivator FOXO1/PGC1α complex. In line with our results, Stein and Tschudy [18] reported clinical improvement in patients when small amounts of insulin were administered together with carbohydrates. More recently, Handschin et al. confirmed that the combination of glucose and insulin causes a more potent inhibition of ALAS1 than the administration of glucose alone [10]. Oliveri et al. (2012) also found hepatic *alas1* down-regulation concomitant with an activation of the phosphoinositide 3-kinase/Akt pathway and subsequent reduction of nuclear FOXO1-PGC-1α complex levels [45]. 

The advantage of our experimental Ins-ApoAI protein is that the apolipoprotein A-I moiety promotes liver targeting [20], thus promoting quicker and sustained repression of hepatic *alas1* expression. One important finding in our study is the repressive effect of the co-administration of glucose and Ins-ApoAI to counteract direct hepatic *alas1* gene induction modulated by barbiturate administration. The co-administration of glucose with fast-acting insulin was unable to modify urine porphyrin precursor excretion in an ongoing attack (treatment study). A possible drawback of the use of fast-acting insulin could be the iatrogenic induction of hypoglycemia. In these circumstances, glucagon secretion and *pgc-1α* are stimulated, and therefore, the effect obtained would be the re-induction of hepatic *alas1*. To avoid this effect in our study, AIP mice co-injected with fast-acting insulin received twice as many glucose doses as those treated with Ins-ApoAI, which would lead to an increased risk of weight gain if the protocol were repeated regularly.

In a preventive study, recurrent administration of glucose for ten days in AIP mice fed ad libitum partially protected against ALA accumulation induced after phenobarbital challenge. However, this reduction did not translate into a positive effect on the behavior of AIP mice because all the animals showed the same degree of pain and motor disability as control AIP mice. Of interest, administration of glucose with two doses of Ins-ApoAI improved pain and motor coordination, although it was unable to protect against heme precursor accumulation. Probably, ApoAI-induced mitochondrial biogenesis in the liver (as shown in Figure 5a and previously reported in [46]), together with increased metabolite supply could promote increased energy production that further improves behavioral parameters in AIP mice.

In the fasted state, the liver is a primary energy source and secretes glucose through both the breakdown of glycogen (glycogenolysis) and de novo glucose synthesis (gluconeogenesis) [47,48]. The hepatic G6Pase enzyme plays an important role in blood glucose homeostasis, and its expression is highly up-regulated during starvation. In fasted WT mice, glucose administration quickly normalized its up-regulation, while in the livers of AIP mice *g6pase* gene overexpression remained. Given that AIP mice showed reduced fat accumulation in vWAT and low levels of circulating triglycerides when compared to WT mice, all these data together suggest that the adipose-liver axis is an important source of energy supply for the rest of the organs in fasted AIP mice, but also that glucose secretion reduces the metabolite availability for the TCA cycle in the hepatocytes. Of interest, the administration of glucose and exogenous insulin in AIP mice increased circulating triglyceride levels as an energy source in non-adipose tissues while reversing hepatic *g6pase* overexpression, thus reducing the hepatic glucose supply.

During the phenobarbital-induced acute attack, there is a large loss of hepatic succinyl-CoA and glycine to produce ALA and PBG, which are rapidly excreted in the urine [49]. Thus, preventive treatment with glucose and insulin would increase hepatocyte metabolite availability that compensates for the loss of succinyl-CoA. In addition, another advantage of using a liver-targeted insulin is the increased hepatic glucose availability for its incorporation into the TCA cycle through pyruvate, enhanced mitochondrial biogenesis, and improved beta-oxidation that increases the energy status in the liver of AIP mice and reduces the porphyrinogenic effects of phenobarbital administration.

## 5. Conclusions

A high prevalence of hyperinsulinemia and IR was observed in Spanish patients carrying a mutation in the *PBGD* gene who had either stable (without clinical outcomes associated with acute porphyria) or asymptomatic disease (AIP-ASHE). The treatment study in AIP mice provides a proof-of-concept for a rapid and sustained repressive effect of Ins-ApoAI on hepatic *alas1* transcription during fasting and acute attacks induced by barbiturate administration. The prophylactic administration of this fusion protein, although associated with behavioral improvements, showed lower effectiveness to reduce porphyrin precursor accumulation compared with a direct *ALAS* inhibitor [50] or with therapies aimed at restoring hepatic PBGD expression [32,51]. Nevertheless, and given the anti-obesity effect associated with an increase of energy expenditure of the ApoAI moiety [52], further studies are needed to evaluate the effect of ApoAI on patients with AIP who are overweight and/or with metabolic syndrome associated with excessive carbohydrate intake or sedentary habits.

## Figures and Tables

**Table 1:** Demographic and clinical characteristics of the study population.

	CV (n=55)	AIP (n=44)	AIP-SD (n=18)	AIP-ASHE (n=18)	AIP-AD (n=8)	*p.* CV vs AIP
**Woman (n)** **(%)**	43 78.2%	36 81.8%	13 72.2%	15 83.3%	8 100%	0.8
**Age (y)** **(range)**	39.2 ± 12.9 17–68	42 ± 14.1 17–68	44.3 ± 16.3 17–68	44.5 ± 11.2 22–63	33 ± 10.9 17–46	0.15
**Body weight (kg)** **(range)**	65.3 ± 11.7 40–92	63.6 ± 16.3 30–98	65.7 ± 17.3 43–97	66.8 ± 16.1 45–98	51.9 ± 9.6 30–59	0.58
**HOMA index** **(range)**	1.64 ± 0.74 0.56–3.53	2.22 ± 1.23 0.68–6.07	2.47 ± 1.56 0.88–6.07	2.16 ± 0.92 0.8–4.1	1.80 ± 0.82 0.68–2.93	0.008
**Serum glucose (mg/dl)** **(range)**	89.2 ± 8.57 68.0–111.5	93.7 ± 9.40 71.0–125.0	95.7 ± 9.95 71.0–113.0	92.8 ± 10.04 81.0–125.0	91.3 ± 6.16 83.0–99.0	0.015
**Serum insulin (µU/dl)** **(range)**	7.55 ± 3.23 2.63–14.9	9.48 ± 5.13 1.8–26.5	10.4 ± 7.03 1.8–26.5	9.27 ± 3.23 3.7–13.9	7.9 ± 3.38 3–11.5	0.032
**Metabolic Sd. (nº)** **(%)**	1/55 1.82%	3/44 6.82%	0/18 0%	1/18 5.6%	2/8 25%	0.333
**Sedentary lifestyle (nº)** **(%)**	22 of 49 * 44.89%	9/44 20.45%	3/18 16.7%	4/18 22.2%	2/8 25%	0.012
**BMI (kg/m^2^)** **(range)**	22.4 ± 3.87 16.7–32.98	24.2 ± 5.88 11.9–36.4	24.9 ± 5.70 16.2–36.4	25.8 ± 5.99 16.7–36.3	19.14 ± 3.27 11.9–22.5	0.99
**ALA (** **μ** **g/mg creat.)** **(range)**	4.97 ± 0.24 3.2–5.0	9.54 ± 10 0.98–57	4.48 ± 1.17 1.3–5.0	9.63 ± 6.60 0.98–25	20.7 ± 17.3 3.3–57	0.003

Data represent mean ± SD (range), except counts of women and individuals with metabolic syndrome and sedentary lifestyle (percentage of total). Data were analyzed using a two-tailed Student’s *t* test on total AIP cases (n = 44) versus matched control volunteers (n = 55) and differences in frequency distribution were analyzed using Fisher’s exact test. CV: control volunteers; AIP: acute intermittent porphyria; AD: cases with active disease; SD: cases with stable disease; ASHE: asymptomatic high excreters patients. Normal urinary levels are <4.64 mg ALA/g creat. (<4 mmol ALA/mol creat.) and <3 mg PBG/g creat. (<1.5 mmol PBG/mol creat.). * There were missing data for six patients.

## Data Availability

The data presented in this study are available on request from the corresponding author.

## Supplementary Materials

The following are available online at https://www.mdpi.com/2227-9059/9/3/255/s1. Figure S1: Hepatic expression of the *alas1* gene in fasted animals treated with glucose, and glucose with a fast-acting insulin or an experimental liver-targeted insulin (Ins-ApoAI). To explore in the animal model the matched dose of insulin and glucose to obtain a treatment effect, in other words, to mimic the “glucose/insulin clamp”, male AIP mice between 12 and 16 weeks old were injected with glucose combined with different concentrations of fast-acting insulin (180 µg/kg or 18 µg/kg; dose eq. to 4.55 or 0.45 μg of pure insulin/mouse) or Ins-ApoAI (90, 30, or 10 µg/kg; dose eq. to 2.3, 0.77 or 0.26 μg of pure insulin/mouse) after 15 h of fasting. The protocol is shown in Figure 2a. At the end of the study, the hepatic expression of the *alas1* gene was measured. The AIP mice that received higher doses of fast-acting insulin (180 µg/kg) showed very low glycemia during the 6 h of study despite being injected with repeated doses of glucose. Hepatic *alas* gene was overexpressed compared to the glucose-treated control. Given that hypoglycemia probably induced the opposite effect than was expected, the results of this group were eliminated from the graph. Data are the mean ± s.d. of five mice per group. Comparisons were performed by one-way ANOVA followed by a Bonferroni post-test. ***, *p* < 0.001 vs. control fasted AIP mice (grey column). *Alas1*: *aminolevulinate synthase 1*. Ins-ApoAI: the fusion protein of a single chain insulin and apolipoprotein A-I; Ins: fast-acting insulin. Supplementary Figure S2: Gene expression profiles of key genes involved in heme synthesis and gluconeogenesis in 15-h-fasted mice fed with glucose with or without exogenous insulin. Fold change expression of the main genes involved in the hepatic regulation of (A) *alas1*, (B) *g6pase*, and (C) *pepck* were analyzed in 15-h-fasted mice and 2.5 h and 6 h after intraperitoneal administration of glucose (2 mg/kg) combined with subcutaneous administration of fast-acting insulin (18 µg/kg, dose eq. to 0.45 μg of pure insulin/mouse) or Ins-ApoAI (eq. from 10, 30, and 90 µg/kg; dose eq. to 0.26, 0.77 or 2.3μg of pure insulin/mouse). Mice received an initial intraperitoneal dose of 2 mg/kg of glucose (20% solution) and supplementary doses when close to hypoglycemic values (<70 mg/dL). Glycemia was measured at 30 min intervals, starting 5 min post-initial dose. Mice treated with Ins-ApoAI showed blood glucose kinetics similar to the control glucose group and received a total of three glucose doses (every 2 h among the 6 h of study). Serum glucose quickly decreased in AIP mice treated with fast-acting insulin in which one dose per hour was necessary to avoid hypoglycemia. Data are mean ± s.d. of five mice per group. Comparisons were performed by one-way ANOVA followed by Bonferroni post-test. *, *p* < 0.05, **, *p* < 0.01; ***, *p* < 0.001 vs. well-fed mice. *Alas1*: *aminolevulinate synthase 1; g6pase*: *glucose 6-phosphatase; pepck*: *phosphoenolpyruvate carboxykinase*; Glu: Glucose; Ins-ApoAI: the fusion protein of a single chain insulin and apolipoprotein A-I; Ins: fast-acting insulin. Supplementary Figure S3: (A) Kinetics of total pAkt/Akt ratio in the liver of AIP mice at different times after glucose or co-administration of glucose and Ins-ApoAI. (B) Representative western blot of pAkt/Akt ratio in 15-h-fasted AIP mice at different times post-treatment. Immunodetection of relevant proteins was determined on PVDF/nitrocellulose membranes. The membranes were blocked (5% milk for Akt, and 5% BSA for GAPDH and pAkt) for 1 h at room temperature and incubated at 4 ºC overnight with the corresponding primary antibody for anti-AKT (1:1000 dilution, #9272, Cell Signaling, MA, USA), anti-GAPDH (1:10.000 dilution, #2118, Cell Signaling) and anti-phosphorylated-AKT (1:1000 dilution, #9271S, Cell Signaling). Mice treated with glucose and Ins-ApoAI showed a significant increase at 10 min, with maximum Akt phosphorylation at 30 min. Quantification of bands was performed using the Image J program in a total of four different Western blots. The normalization of the values was undertaken on the same baseline group of mice. Supplementary Figure S4: Representative liver sections of (A) a non-injected AIP mouse, (B) an AIP mouse treated with glucose and Ins-ApoAI and (C) an AIP mouse co-administered with glucose and fast-acting insulin. Scale ×80.
