# Supplementary figures and images for: High Prevalence of Insulin Resistance in Asymptomatic Patients with Acute Intermittent Porphyria and Liver-Targeted Insulin as a Novel Therapeutic Approach
Source: Biomedicines. 2021 Mar 5;9(3):255. doi: 10.3390/biomedicines9030255 (PMC8002016; doi:10.3390/biomedicines9030255)

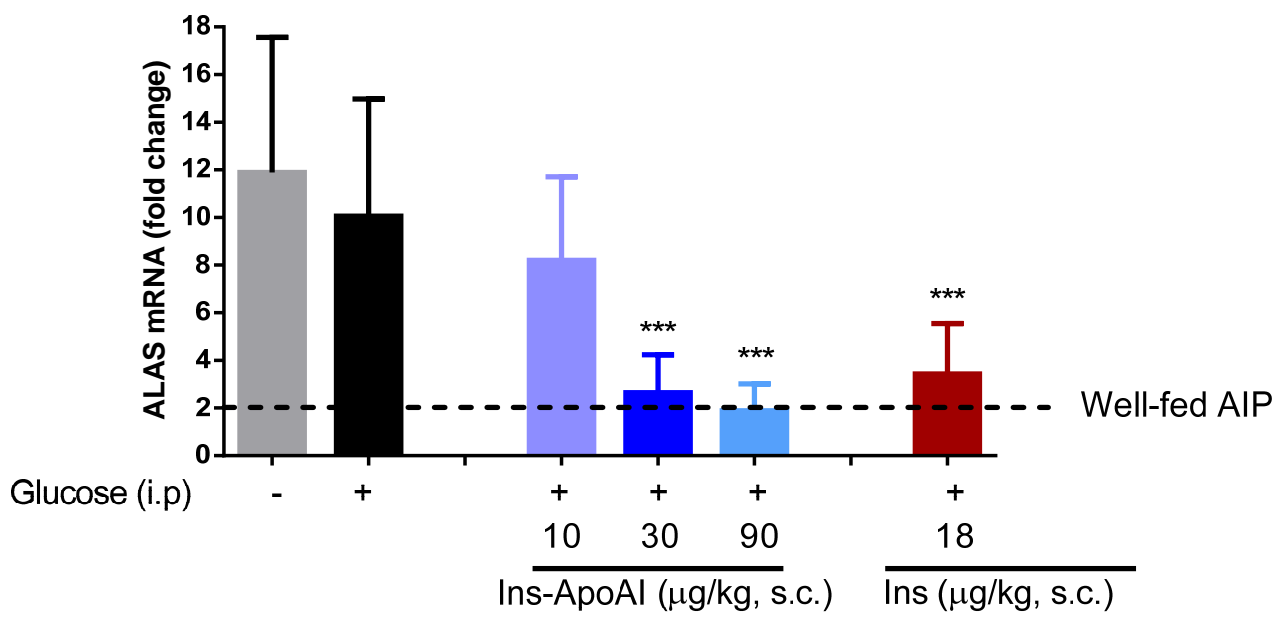

Supplement: Supplementary file 1 [file biomedicines-09-00255-s001.zip › Supplementary Figure 1 Solares et al.pdf]

## A) WT mice

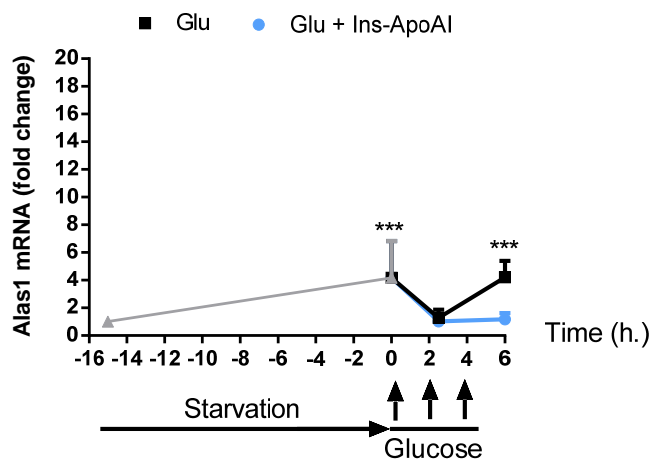

## AIP mice

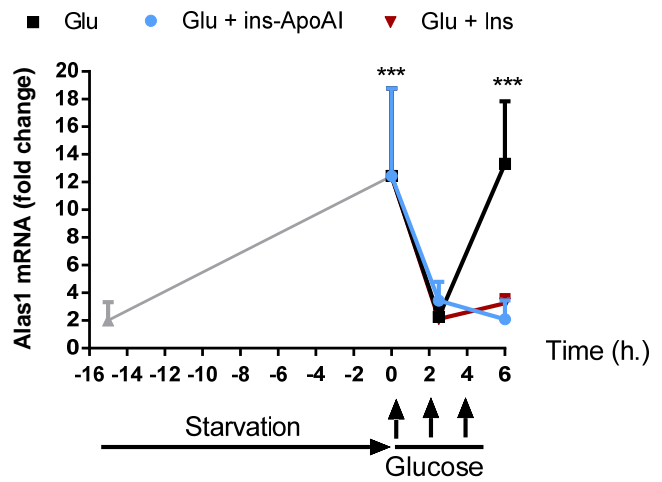

## B)

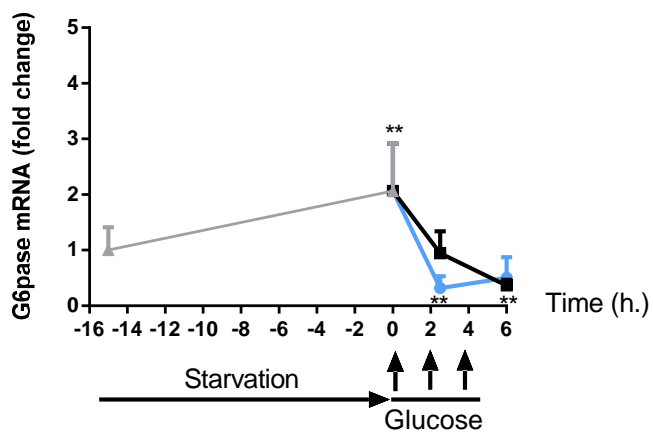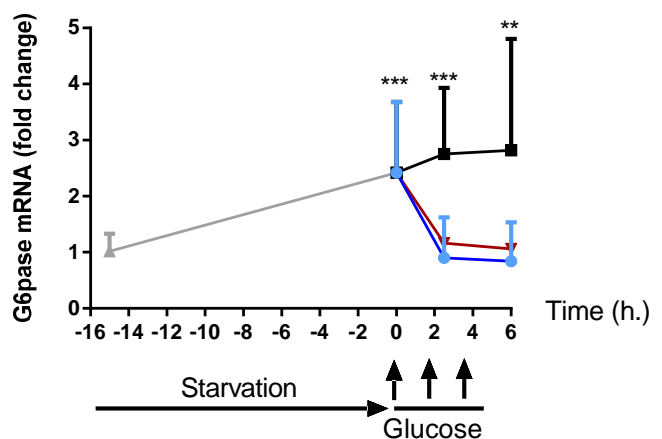

## C)

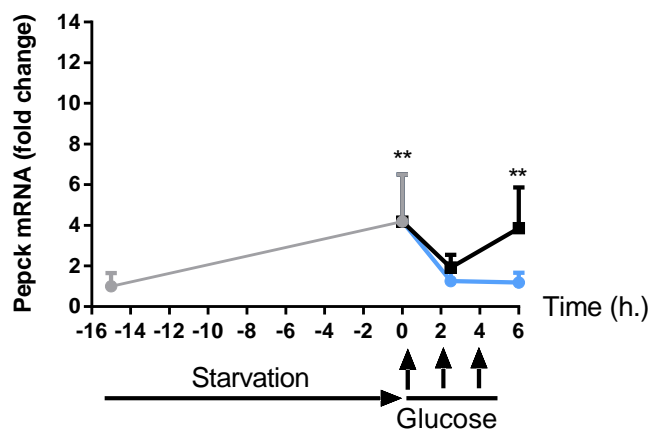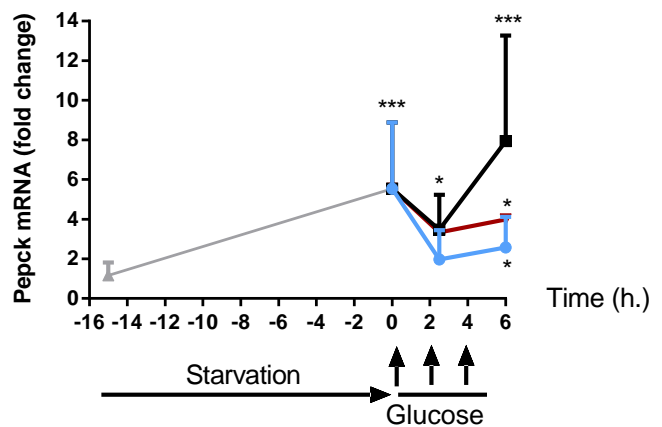

Supplement: Supplementary file 1 [file biomedicines-09-00255-s001.zip › Supplementary Figure 2 Solares et al.pdf]

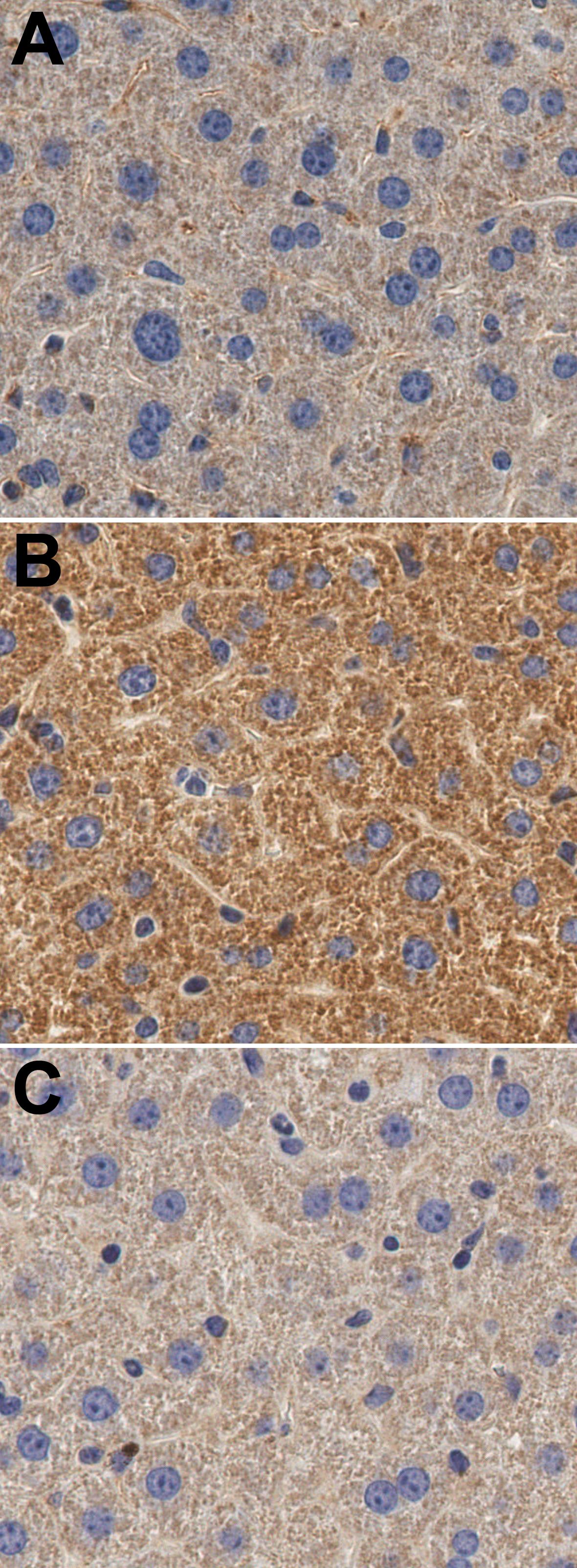

Supplement: Supplementary file 1 [file biomedicines-09-00255-s001.zip › Supplementary Figure 3.tif]
